# The Spatial Correlation and Anisotropy of β-(Al_x_Ga_1−x_)_2_O_3_ Single Crystal

**DOI:** 10.3390/ma16124269

**Published:** 2023-06-08

**Authors:** Liuyan Li, Lingyu Wan, Changtai Xia, Qinglin Sai, Devki N. Talwar, Zhe Chuan Feng, Haoyue Liu, Jiang Jiang, Ping Li

**Affiliations:** 1 Center on Nanoenergy Research, Guangxi Colleges and Universities Key Laboratory of Blue Energy and Systems Integration, Carbon Peak and Neutrality Science and Technology Development Institute, School of Physical Science & Technology, Guangxi University, Nanning 530004, China; 2 State Key Laboratory of Featured Metal Materials and Life-Cycle Safety for Composite Structures, Nanning 530004, China; 3 Key Laboratory of Materials for High Power Laser, Shanghai Institute of Optics and Fine Mechanics, Chinese Academy of Sciences, Shanghai 201800, China; 4 Department of Physics, University of North Florida, Jacksonville, FL 32224, USA; 5 Department of Electrical & Computer Engineering, Southern Polytechnic College of Engineering and Engineering Technology, Kennesaw State University Marietta, Marietta, GA 30060, USA; 6 Hangzhou Institute of Optics and Fine Mechanics, Hangzhou 311421, China

**Keywords:** aluminum gallium oxide, Raman scattering spectroscopy, spatial correlation model, anisotropy of phonon modes

## Abstract

The long-range crystallographic order and anisotropy in β-(Al_x_Ga_1−x_)_2_O_3_ (x = 0.0, 0.06, 0.11, 0.17, 0.26) crystals, prepared by optical floating zone method with different Al composition, is systematically studied by spatial correlation model and using an angle-resolved polarized Raman spectroscopy. Alloying with aluminum is seen as causing Raman peaks to blue shift while their full widths at half maxima broadened. As x increased, the correlation length (CL) of the Raman modes decreased. By changing x, the CL is more strongly affected for low-frequency phonons than the modes in the high-frequency region. For each Raman mode, the CL is decreased by increasing temperature. The results of angle-resolved polarized Raman spectroscopy have revealed that the intensities of β-(Al_x_Ga_1−x_)_2_O_3_ peaks are highly polarization dependent, with significant effects on the anisotropy with alloying. As the Al composition increased, the anisotropy of Raman tensor elements was enhanced for the two strongest phonon modes in the low-frequency range, while the anisotropy of the sharpest Raman phonon modes in the high-frequency region decreased. Our comprehensive study has provided meaningful results for comprehending the long-range orderliness and anisotropy in technologically important β-(Al_x_Ga_1−x_)_2_O_3_ crystals.

## 1. Introduction

Gallium oxide (Ga_2_O_3_), with a wider bandgap energy of 4.8 eV material in the deep ultraviolet (UV) spectrum, is a transparent semiconductor. Its transparency in the UV region has allowed applications such as solar-blind photodetectors and deep-UV transparent contacts. The material with a high breakdown electric field (6–8 MV/cm), large thermal conductivity, low electron effective mass, and the ability to achieve controllable n-type doping with stable chemical properties has made Ga_2_O_3_ promising for engineering the next-generation electronic devices, especially in power-electronics, viz., rectifiers, metal-semiconductor field-effect transistors (MESFETs), metal oxide semiconductor field-effect transistors (MOSFETs), deep ultraviolet detectors, Schottky barrier diodes, etc. [1,2,3,4]. In many other technological applications, it is required to have materials to serve as a dielectric with band gap energy even larger than Ga_2_O_3_ to form heterostructures for carrier confinement and to enable optoelectronics deeper into the UV region. The Al_2_O_3_ with a band gap of 8.82 eV in the corundum (sapphire) phase is considered an excellent candidate for alloying it with Ga_2_O_3_ to create novel β-(Al_x_Ga_1−x_)_2_O_3_. The β-(Al_x_Ga_1−x_)_2_O_3_ has a smaller bond length, which makes the atomic gap smaller and the band gap larger. By carefully increasing Al contents, the band gap of β-(Al_x_Ga_1−x_)_2_O_3_ has the potential to increase from 4.8 eV to 6 eV. This material with higher critical field strength is considered suitable for power electronics devices and shorter cut-off wavelength for optoelectronic devices [5,6,7]. In a recent study [8], the drain current of (Al_0.08_Ga_0.92_)_2_O_3_/Ga_2_O_3_ MODFET (with a gate voltage of +3 V) can be 12 mA mm^−1^, and the on/off current ratio is about 10^4^. The breakdown voltage of the (Al_0.16_Ga_0.84_)_2_O_3_-channel MESFETs (with a drain-to-gate spacing of 20 μm) can reach 940 V, which is higher than that of β- Ga_2_O_3_. In addition, β-(Al_x_Ga_1−x_)_2_O_3_ can be used as a barrier layer for β-(Al_x_Ga_1−x_)_2_O_3_/β-Ga_2_O_3_ HEMTs. The theoretical value of 2DEG mobility for β-(Al_x_Ga_1−x_)_2_O_3_/β-Ga_2_O_3_ interface can reach 500 cm^2^/V s, which is much higher than that of the bulk β-Ga_2_O_3_ [9]. It means that (Al_x_Ga_1−x_)_2_O_3_ has great potential for high-power applications.

In early research, it was found that the internal changes of materials often change their physical properties, thus affecting their application in devices [10,11]. In the process of alloying Ga_2_O_3_ with Al_2_O_3_, the microstructure and physical properties of the crystal are modified due to the change of atomic radius, lattice structure, and the formation of new compositions and defects during the crystal growth. In an earlier theoretical study, Peelaers et al. [12] have shown that the pseudo-cubic lattice constant of β-(Al_x_Ga_1−x_)_2_O_3_ alloys decrease linearly by increasing Al composition x, while the bandgap increases exhibiting significant bowing. Moreover, it has been suggested that Al atoms preferentially occupy the octahedral positions to form the monoclinic structure for x < 0.71. While studying the electronic and optical properties, Ma et al. [13] have noticed that Al doping in β-Ga_2_O_3_ leads to the formation of O interstitial defects. Kim et al. [14] have suggested that the oxygen vacancies and grain boundary-related defects increase in (Al_x_Ga_1−x_)_2_O_3_ with Al composition exceeding 0.6 at %. Bhuiyan et al. [15] have studied the phase stability and phase transition of (Al_x_Ga_1−x_)_2_O_3_ films grown by MOCVD in the wide Al composition range. The results have shown that the strain caused by the doping of Al induces rotation of the β phase (Al_x_Ga_1-x_)_2_O_3_ crystal phase domain and promotes the formation of γ phase (Al_x_Ga_1−x_)_2_O_3_. Hilfiker et al. [16] reported the dielectric function tensor of β-(Al_x_Ga_1−x_)_2_O_3_ (x ≤ 0.21) films exhibiting strong anisotropy and inter-band transitions in the energy range of 1.5–9.0 eV. As the Al concentration increases, the band gap of the thin films increases, and the anisotropy increases.

In our previous work [17], we have reported crystal structure distortions by studying the temperature dependence of Raman shifts in β-(Al_x_Ga_1−x_)_2_O_3_ single crystals with x = 0, 0.1, 0.2. Certainly, with distortion, the lattice vibrations and anisotropy of the crystal will change. However, the changes in the long-range order and the anisotropy of lattice vibration caused by alloying with aluminum in β-(Al_x_Ga_1−x_)_2_O_3_ are still unclear. The purpose of this work is to study the spatial correlation of Raman phonon modes in β-Ga_2_O_3_ single crystal and its alloyed β-(Al_x_Ga_1−x_)_2_O_3_ single crystals with different Al compositions, x (≡0.0, 0.06, 0.11, 0.17, 0.26) by using a spatial correlation model. The long-range crystallographic order within the crystal and the symmetry of its sublattice structure are reflected by the correlation length (CL). In addition, the angle-resolved polarized Raman spectroscopy (ARPRS) was carried out to observe the angle dependence of β-(Al_x_Ga_1−x_)_2_O_3_ alloys in different polarization configurations. Combining with the Raman selection rules, we have analyzed the Raman tensor elements of different alloyed samples and revealed the intrinsic anisotropy of differential polarizability.

## 2. Material Growth and Characterization Methods

The five β-(Al_x_Ga_1−x_)_2_O_3_ single-crystal samples are grown by using the optical floating zone method, with aluminum composition x < 0.3. Energy Dispersive Spectroscopy (EDS) (XMax20/C-Nano, Sigma-500 Zeiss, Oberkochen, Germany) is used to analyze the composition of aluminum fraction in these samples. The total quantity error of EDS is ≤3%. The surface scanning mode has been set with a scanning range of 2 mm × 2 mm. The samples are carefully analyzed using a high-resolution x-ray diffraction (HR-XRD) spectroscopy (model SMARTLAB 3kW, Rigaku, Tokyo, Japan) by employing a Cu target excitation source for assessing their crystalline quality. The accuracy of XRD is 0.01°.

**Figure 1:**
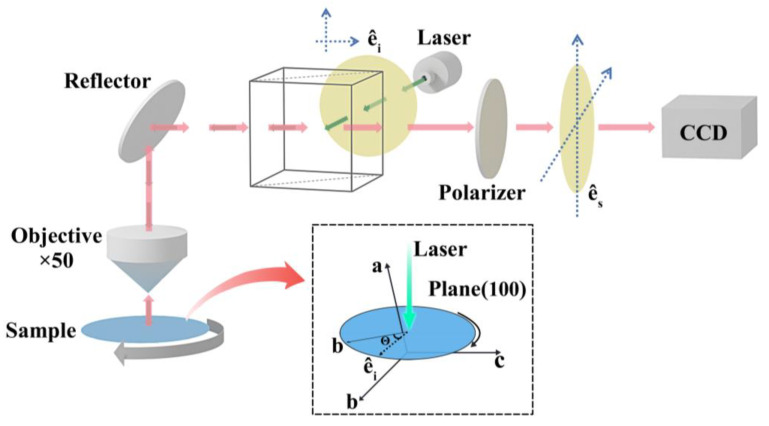
Schematic diagrams of angle-resolution Raman experimental configuration; ê_i_ and ê_s_ denote the unit polarization vectors of the incident and scattered light, respectively.

Raman scattering spectroscopy measurements are conducted by using the Zolix Raman microscopic spectrograph (model Finder One), which is employed by using a 532 nm wavelength laser as an excitation source. The test precision of Raman spectrometer is ≤2 cm^−1^. The temperature-dependent studies between 80 K and 800 K are recorded with a Linkam INSTEC temperature-changing device. The temperature error is ±0.1 K. In the original Raman scattering measurement system, a polarizer is placed in front of the detector. By rotating the b-axis direction of the sample, the angular dependence from 0° to 360° is tested with an angle step of 5°. The angle error bar in ARPRS is ±0.05°. The experimental configuration is shown in Figure 1. The parallel-polarization and cross-polarization configurations are realized by adjusting the polarizer angles between 0° and 90° in the optical detecting path. The sample placement follows the notion that the polished surface of the sample is perpendicular to the direction of light propagation.

## 3. Results and Discussion

### 3.1. Crystal Structure and Composition

The results for the β-(Al_x_Ga_1−x_)_2_O_3_ samples analyzed qualitatively and quantitatively by using EDS are displayed in Figure S1. For every sample, the calculated atomic percentage reported in Table 1 is conducted by considering the peak area ratio of each feature. The Al/(Al + Ga) molar ratios in five samples are 0.0, 0.06, 0.11, 0.17, and 0.26, respectively. For convenience, the β-Ga_2_O_3_, β-(Al_0.06_Ga_0.94_)_2_O_3_, β-(A_0.11_Ga_0.89_)_2_O_3_, β-(Al_0.17_Ga_0.83_)_2_O_3_, and β-(Al_0.26_Ga_0.74_)_2_O_3_ samples are marked as S_0_, S_1_, S_2_, S_3_, and S_4_, respectively. The results of optical transmission spectra are displayed in Figure S2 for the five samples. The bandgaps obtained for samples S_0_, S_1_, S_2_, S_3_, and S_4_ by linear extrapolation using Tauc’s method are 4.708, 4.761, 4.885, 4.982, and 5.63 eV, respectively (Figure S2).

**Figure 2:**
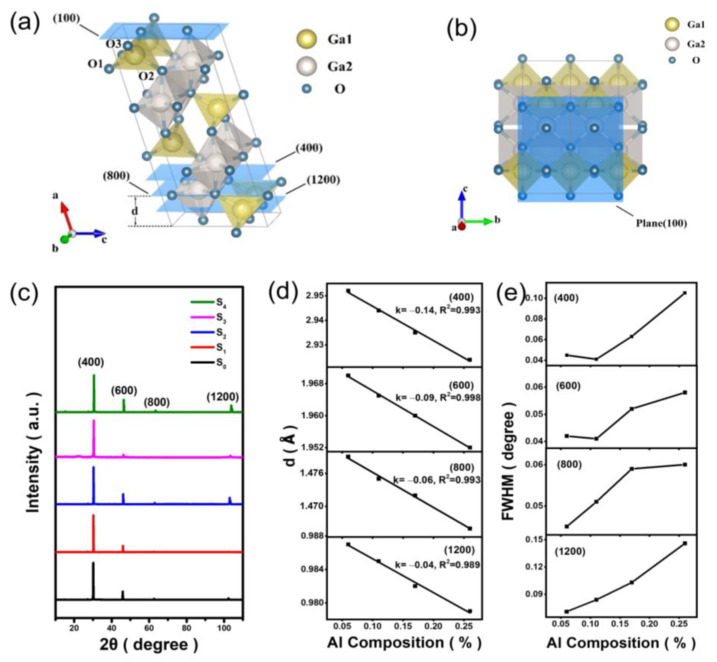
(**a**) The crystalline structure and (**b**) (100) plane of β-Ga_2_O_3_; (**c**) XRD patterns of S_0_, S_1_, S_2_, S_3_, and S_4_; (**d**) the interplanar crystal spacing; (**e**) FWHM for (Al_x_Ga_1−x_)_2_O_3_ samples in dependence on the Al concentration. The variable k is the slope factor. R^2^ is the coefficient of determination that reflects the fitting effect. The closer the value is to 1, the better the model fitting degree is, and the smaller the error is.

The β-Ga_2_O_3_ single crystal has a monoclinic structure formed by the stacking of two GaO_4_ tetrahedra and two edge-sharing GaO_6_ octahedra, as shown in Figure 2a,b. The XRD spectroscopy results displayed in Figure 2c have clearly shown four diffraction peaks corresponding to the (400), (600), (800), and (1200) crystal planes of β-Ga_2_O_3_, referring to the β-Ga_2_O_3_ XRD JCPDS card (04-003-1858). The five samples have exhibited well-defined monoclinic structures. From the Bragg’s Equation (1)

(1) 2dsin⁡θ=nλ, (n=1, 2, 3……),

it is possible to obtain the variation trends in the interplanar crystal spacing d with the aluminum composition, x. From Figure 2d,e, one may notice that by increasing x, the interplanar crystal spacing decreases while the FWHM broadens. As the volume size of Al atoms is smaller than that of Ga, the average bond length of Al-O is, therefore, shorter than Ga-O bonds. The replacement of Ga by Al atoms caused the (Al_x_Ga_1−x_)_2_O_3_ unit cell volume to decrease [18,19]. This has resulted in the decrease of interplanar crystal spacing (d). Linear fitting of the interplanar spacing has shown that the degree of change in d with aluminum composition varied differently for different crystal planes. The absolute values of the slopes (|k|) obtained by fitting the diffraction peaks of the (400), (600), (800), and (1200) planes were 0.14, 0.09, 0.06, and 0.04, respectively. With the increase of Miller index, the influence of aluminum composition on the interplanar crystal spacing gradually decreases.

### 3.2. Raman Scattering Spectroscopy

**Figure 3:**
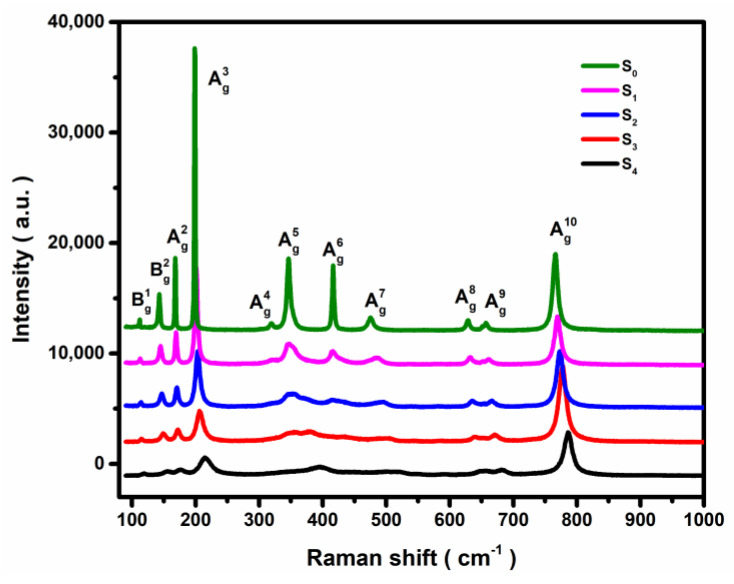
RT Raman scattering spectra of S_0_, S_1_, S_2_, S_3_, and S_4_.

The room-temperature (RT) Raman scattering spectra of the five samples, excited by a 532 nm laser (perpendicular to the (100) plane) source, are shown in Figure 3. All five samples, S_0_, S_1_, S_2_, S_3_, and S_4_, have revealed 10 Raman active phonon features ($B_{g}^{2}$, $A_{g}^{2}$, $A_{g}^{3}$, $A_{g}^{4}$, $A_{g}^{5}$, $A_{g}^{6}$, $A_{g}^{7}$, $A_{g}^{8}$, $A_{g}^{9}$, and $A_{g}^{10}$) in the frequency range of 100~1000 cm^−1^_._ The results agreed very well with those reported in the literature [20,21,22]. One must note that the modes belonging to the $B_{g}^{2}$, $A_{g}^{2}$, and $A_{g}^{3}$ are associated with the vibration and translation of the [GaO_6_]-[GaO_4_] chains; the $A_{g}^{4}$, $A_{g}^{19}$, $A_{g}^{6}$, and $A_{g}^{7}$ modes are related to the deformation of the [GaO_6_] octahedron while $A_{g}^{7}$, $A_{g}^{8}$, and $A_{g}^{10}$ phonons are linked to the symmetric stretching and bending vibrational modes of the [GaO_4_] tetrahedron [17,23]. The perusal of Figure 3a reveals that with an increase in Al composition, the Raman intensity of each band has weakened. The shape of every peak in the mid-frequency region is distorted due to alloying, and the features have no longer behaved as the normal Lorentz types. The XRD results indicate the lattice distortion is induced by the substitution of Al for the Ga atoms. This reduced the crystal lattice symmetry by weakening the bond strength and caused the Raman active modes to shift with declining intensity [24].

**Figure 4:**
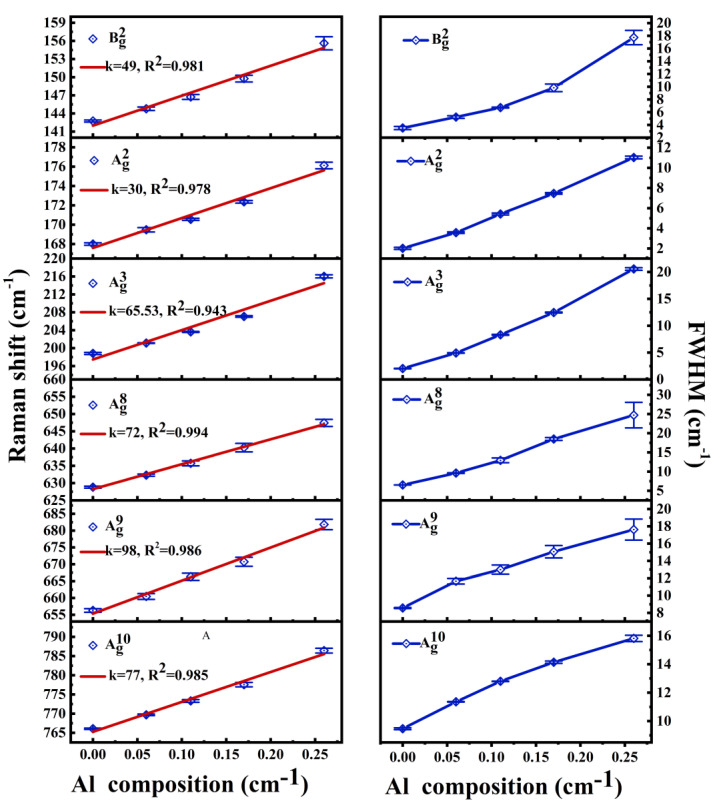
The Raman shift (**a**) and the FWHM (**b**) for (Al_x_Ga_1−x_)_2_O_3_ samples versus Al composition.

The Raman shifts and full width at half maximum (FWHM) for (Al_x_Ga_1−x_)_2_O_3_ samples are displayed in Figure 4 as a function of Al composition, x. From Figure 4a, we have noticed that increasing x caused a blue shift to the Raman phonon peaks. The linear fit of Raman shifts as a function of composition indicated the slopes of $B_{g}^{2}$, $A_{g}^{2}$, $A_{g}^{3}$, $A_{g}^{8}$, $A_{g}^{9}$, and $A_{g}^{10}$ modes as 48, 29, 64, 73, 99, and 77, respectively. The slope values corroborate those reported by Kranert et al. [21]. Earlier, Janzen et al. [20] calculated the contributions of different atoms to the phonon energies. By comparison, we found that the modes with a larger influence of O atoms on the total phonon energy exhibit larger k values. This is related to the force constants between the molecular bonds. As the average bond length of Al-O is shorter than those of the Ga-O bonds, alloying with Al would, therefore, cause the force constants between Al-O bonds to strengthen [18,25]. The decrease of the atomic mass and increase in the force constant are the main reasons for the observed blue shifts to the Raman peaks in the β-(Al_x_Ga_1−x_)_2_O_3_ alloys [23,26]. The mass change comes from the difference in masses of the substituted Al for Ga atoms, while the change in force constant is caused by the Al-O bonds generated after the substitution of Al atoms. The larger contribution of O means higher involvement of the Al-O bonds. Obviously, the results have indicated the contribution of force constant change between Al-O bonds to the blue shift of Raman peaks is stronger than the mass change of the atoms. Interestingly, the contribution of O atoms to the $B_{g}^{2}$ mode is similar to those of the $A_{g}^{2}$ mode: the slope k of the $B_{g}^{2}$ mode is nearly twice that of the $A_{g}^{2}$ mode. This may be related to the fact that the A_g_ mode oscillates in the b-plane while the B_g_ mode vibrates perpendicular to the b-plane [26]. In our work, the light path is perpendicular to the (100) plane, and under the same comparative configuration, the component of the vibrational amplitude of B_g_ mode in the (100) plane is larger. In addition, it is also noticed that the Al composition x has a smaller effect on the low-frequency modes as compared to the high-frequency phonons. From the earlier studies [23,24], the low-frequency modes are suggested as mainly originating from the vibrations of the tetrahedral-octahedral chains, while the high-frequency modes involved the atomic vibrations of the tetrahedral units. As compared to the vibrations of the chains, the impact of doping on the phonon energy is more obvious in the modes related to the tetrahedral units.

Again, the perusal of Figure 3 and Figure 4b revealed that the FWHM of Raman peaks for different modes are broadened with the increase of Al composition, x, especially in the mid-frequency range, where the distortions occurred at varying degrees, and the main Lorentzian shapes are lost. This may be related to the selective substitution of Al atoms, which preferably substitute for the octahedral Ga atoms as compared to the tetrahedral Ga atoms [21,27]. Unlike the trends of blue shift, the FWHM (cf. Figure 4b) reflects the disorder in the observed phonons. It can be noticed that the low-frequency phonons are more sensitive to the Al doping concentration, with the turning point appearing near x = 0.17. After that, the FWHM begins to deviate from the linear relationship with composition, x. This may be related to the phonon vibration. When the doping amount is sufficient, the atomic substitution reaches a certain degree, and the disorder of phonons increases, which may lead to the formation of new phonon modes. Especially the mid-frequency phonons. This is mentioned in the study of β-(Al_x_Ga_1−x_)_2_O_3_ ceramics by Bhattacharjee et al. [26]. The high-frequency region is only related to the vibration of the tetrahedron, so there is no turning point at x = 0.17. The low-frequency phonon is related to the vibration of the chains, so the FWHM broadening is also affected by the mid-frequency phonons, which is out of the linear relationship. As Compared to the pure β-Ga_2_O_3_ sample, when Al composition reaches close to x = 0.26 in β-(Al_x_Ga_1−x_)_2_O_3_ alloys, the FWHM of $B_{g}^{2}$, $A_{g}^{2}$, and $A_{g}^{3}$ modes broaden (nearly 5 to 10 times) with $A_{g}^{3}$ mode showing the most significant changes. The $A_{g}^{8}$, $A_{g}^{9}$, and $A_{g}^{10}$ modes, however, broaden only 2–3 times at x = 0.26. The $B_{g}^{2}$, $A_{g}^{2}$, and $A_{g}^{3}$ modes involve different types of atoms; therefore, they cause a greater degree of phonon disorder and lead to a significant broadening of the FWHM.

### 3.3. Spatial Correlation of Raman Phonons

Again, the spatial correlation model of Raman scattering spectra can be used to analyze the effects of Al composition, x, on the lattice vibrational modes order of the alloys [28,29,30,31]. As the aluminum composition is increased in our β-(Al_x_Ga_1−x_)_2_O_3_ samples, each Raman peak produces different degrees of asymmetric broadening and causes a blue shift. In a perfect crystal with translational symmetry, the spatial correlation function of phonons is infinite, which follows the selection rule of q = 0. However, due to the lattice distortion and defects in β-(Al_x_Ga_1−x_)_2_O_3_ alloys, the disorder of microstructures increases, the spatial correlation function of the phonon becomes limited, and the q = 0 selection rule is destroyed [30]. According to the CL of the phonons, the perfection of crystallization and disorder of lattice vibration can be analyzed quantitatively.

It is assumed that there exists a spherical region associated with a finite size of the correlation region in the alloy material. Combining with a Gaussian distribution function, the Raman intensity *I*(ω) at a frequency ω can be expressed as the following [32,33,34]:

(2) $$I(\omega )\propto \int_{0}^{1}\exp(-\frac{q^{2}L^{2}}{4})\frac{d^{3}q}{{\left[\omega -\omega \left(q\right)\right]}^{2}+{\left(\frac{\Gamma_{0}}{2}\right)}^{2}}$$

Here, *q* (≡2π/a) represents the magnitude of the normalized wavevector, where a is the lattice constant of the sample. The term *L* in Equation (2) is the CL of Raman mode which represents the range of ordered phonons described by the correlation function. It is commonly used to explain the q-vector relaxation related to finite-size effects and structural disorder. The symbol Γ_0_ is the FWHM of the Raman peak; *ω* represents the angular frequency of phonon vibration; *ω*(*q*) is the dispersion relation of optical phonons, which can be expressed as the following [35,36]:

(3) $$\omega \left(q\right)=A-Bq^{2},$$

where A is the phonon frequency at the center of the Brillouin, and B is the correction parameter for the peak position shift of the Raman peak caused by the surrounding environment.

**Figure 5:**
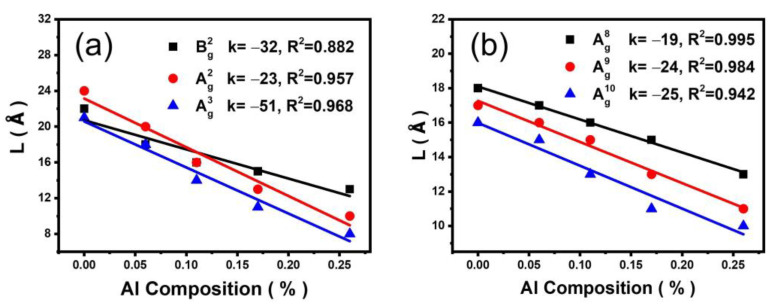
(**a**) The correlation length for $B_{g}^{2}$, $A_{g}^{2}$, $A_{g}^{3}$ phonons, (**b**) $A_{g}^{8}$, $A_{g}^{9}$, and $A_{g}^{10}$ phonons in dependence on the Al composition.

Figure 5 exhibits the relationship between the CLs of the phonon modes and the aluminum composition in the low and high-frequency regions. It can be observed that the CLs decrease with the increase of Al composition, indicating a decrease in the orderliness of the lattice vibrations due to the Al doping. The decrease in orderliness becomes more prominent with the increase of Al composition. The relationship between the CL and the Al composition is linearly fitted. The absolute values of the slope (|k|) of the $B_{g}^{2}$, $A_{g}^{2}$, $A_{g}^{3}$, $A_{g}^{8}$, $A_{g}^{9}$, and $A_{g}^{10}$ modes are 0.42, 0.70, 0.66, 0.24, 0.30, and 0.32, respectively. The modes in the low-frequency region are more sensitive to the Al composition than those of the high-frequency modes. The value |k| of $B_{g}^{2}$ in the low-frequency region is significantly smaller than that of the $A_{g}^{2}$ and $A_{g}^{3}$ modes, while in the high-frequency region, the |k| values of $A_{g}^{8}$, $A_{g}^{9}$, and $A_{g}^{10}$ modes vary significantly. Compared to the chain structures involving multiple sites, the phonons in the high-frequency region are only affected by the atomic substitution in the sublattice related to their vibration resulting in relatively smaller changes in the spatial correlation.

**Figure 6:**
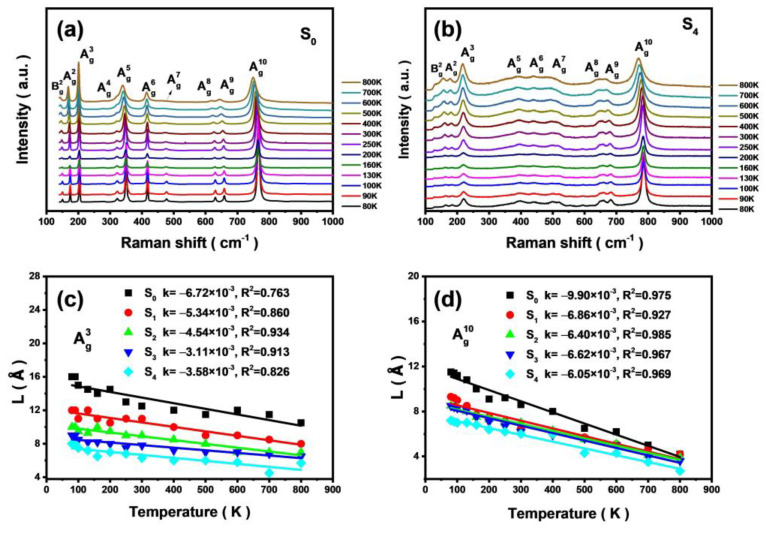
VT Raman scattering spectra of S_0_ (**a**) and S_4_ (**b**); temperature dependence of the correlation length for $A_{g}^{3}$ (**c**) and $A_{g}^{10}$ phonon (**d**).

Figure 6a,b shows the variable-temperature (VT) Raman spectra of S_0_ and S_4_. The VT Raman spectra of S_1_, S_2_, and S_3_ are shown in Figure S3. The values of CLs for $A_{g}^{3}$ (low frequency) and $A_{g}^{10}$ (high frequency) modes at different temperatures are obtained by spatial correlation model fitting to analyze the effects of temperature on the lattice vibration disorder. From Figure 6, one may note that the CLs of the two Raman modes decrease with increasing temperature. As the temperature increases, the disorder in the lattice vibration increases. This can be explained by the fact that as the temperature increases, the thermal motion of the molecule increases, accompanied by lattice expansion [24,37], resulting in enhanced vibrational disorder and shortening the phonon length. With the increase of Al composition, x, the spatial CL range decreases accordingly. This is consistent with the changing trend of the calculated CLs calculated (cf. Figure 6) at room temperature. From the slope of the absolute value(|k|) obtained by linear fitting, it can be noticed that with the increase of Al composition, x, the degree of sublattice order involved in $A_{g}^{3}$ and $A_{g}^{10}$ modes is less affected by temperature. Among them, the difference between the pure β-Ga_2_O_3_ and β-(Al_x_Ga_1−x_)_2_O_3_ alloy samples is particularly obvious. The value of |k| for $A_{g}^{3}$ decreases from 6.72 × 10^−3^ for the undoped sample to 3.11 × 10^−3^ for a sample with Al composition x = 0.17 and then begins to fluctuate slightly. The value of k for $A_{g}^{10}$ decreases from 9.90 × 10^−3^ for the undoped sample to 6.40 × 10^−3^ with an Al composition of x = 0.11 and then begins to fluctuate slightly. This difference is attributed to the fact that the crystal distortion caused by Al doping is partly restored with increasing temperature [17], and the temperature dependence tends to stabilize after reaching a certain doping level. For bulk β-(Al_x_Ga_1−x_)_2_O_3_, doping leads to changes in the microstructure of the material, and the crystal quality of the sample will seriously affect the performance of the device. This paper reveals the relationship between crystal quality, microstructure, and doping concentration in β-(Al_x_Ga_1−x_)_2_O_3_, which provides a reference for the application of β-(Al_x_Ga_1−x_)_2_O_3_ alloy in devices.

### 3.4. In-Plane Anisotropy

Angle-resolved polarized Raman spectroscopy (ARPRS) is utilized to investigate the anisotropic properties of various alloyed samples [38,39,40]. Zhang et al. [41] have successfully used ARPRS to calculate the Raman tensor of β-Ga_2_O_3_, revealing the complexity of the anisotropy of the internal structure of β-Ga_2_O_3_. However, the research on the phonon anisotropy of β-(Al_x_Ga_1−x_)_2_O_3_ has not been reported. In this work, Raman spectra are acquired at different rotation angles of the samples under parallel/cross polarization configurations, where the polarization of the Raman scattered light was parallel/cross to the polarization of the incident light. By rotating the sample, the angle θ between the polarization direction of the incident light and the b-axis of the sample was altered. The experimental setup is shown in Figure 1. The generalized form of the scattering intensity (I) of the ARPRS signal can be mathematically expressed as the following [42,43]:

(4) $$I_{A_{g}}\propto {\left|{\hat{e}}_{i}\cdot R\cdot {\hat{e}}_{s}\right|}^{2}$$

Here, *ê_i_* and *ê_s_* denote the unit polarization vectors of the incident and scattered light, respectively. As the light is incident perpendicular to the sample surface, the incident and scattered light can be represented as *ê_i_* = (sinθ cosθ 0), ${ê}_{s}^{∥}$= (sinθ cosθ 0), and ${ê}_{s}^{⊥}$= (cosθ − sinθ 0). R represents the Raman tensor in the scattering process, and the Raman tensor elements for the *A_g_* and *B_g_* modes of gallium oxide are given as follows [41,44]:

(5) $$R\left(A_{g}\right)=\left(\begin{array}{ccc} b & 0 & d\\ 0 & c & 0\\ d & 0 & a \end{array}\right)$$

(6) $$R(B_{g})=\left(\begin{array}{ccc} 0 & f & 0\\ f & 0 & e\\ 0 & e & 0 \end{array}\right)$$

Here, *a*, *b*, *c*, *d*, *e*, and *f* are the independent Raman tensor elements. To interpret the experimental results more accurately, it is necessary to convert R into a complex second-rank Raman tensor. Therefore, the Raman tensor for β-Ga_2_O_3_ can be expressed as follows:

(7) $$R\left(A_{g}\right)=\left(\begin{array}{ccc} \left|b\right|e^{i\varphi_{b}} & 0 & \left|d\right|e^{i\varphi_{d}}\\ 0 & \left|c\right|e^{i\varphi_{c}} & 0\\ \left|d\right|e^{i\varphi_{d}} & 0 & \left|a\right|e^{i\varphi_{a}} \end{array}\right)$$

(8) $$R(B_{g})=\left(\begin{array}{ccc} 0 & |f|e^{i\varphi_{f}} & 0\\ |f|e^{i\varphi_{f}} & 0 & |e|e^{i\varphi_{e}}\\ 0 & |e|e^{i\varphi_{e}} & 0 \end{array}\right)$$

The phase information of the tensor components is represented by *φ_a_*~*φ_f_*. The angle dependence of the polarized Raman signalcan be described by Equation (4). Substituting the defined *ê_i_* and *ê_s_*, as well as the complex Raman tensor (Equations (7) and (8)), into Equation (4), the ARPRS intensity expression for the plane (100) can be obtained as follows:

(9) $$I_{A_{g}}^{∥}\propto {\left|a\right|}^{2}{sin}^{4}\theta +{\left|c\right|}^{2}{cos}^{4}\theta +2|a||c|{sin}^{2}\theta {cos}^{2}\theta {cos}_{\varphi_{ac}}$$

(10) $$I_{A_{g}}^{⊥}\propto {sin}^{2}\theta {cos}^{2}\theta \cdot ({\left|a\right|}^{2}+{\left|c\right|}^{2}-2\left|a\right|\left|c\right|{cos}_{\varphi_{ac}})$$

(11) $$I_{B_{g}}^{∥}\propto {\left[\left|e\right|\cdot \sin\left(2\theta \right)\right]}^{2}$$

(12) $$I_{B_{g}}^{⊥∥}\propto {\left[\left|e\right|\cdot \cos\left(2\theta \right)\right]}^{2}$$

where *φ* is the phase difference between the two distinct elements of the Raman tensor.

**Figure 7:**
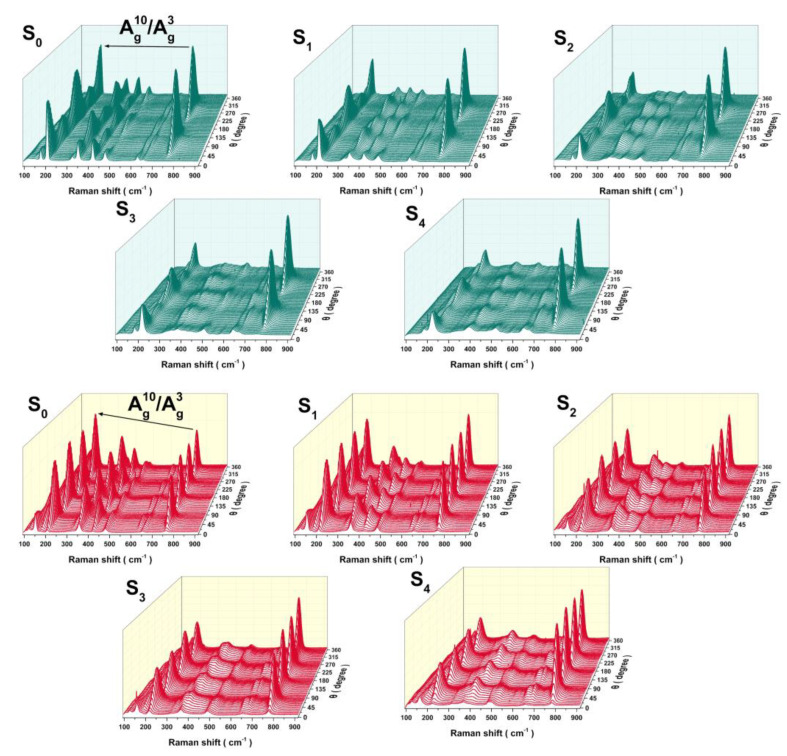
Waterfall plot of the angle-resolved polarized Raman spectra for S_0_, S_1_, S_2_, S_3_, and S_4_ measured in parallel-polarized and cross-polarized configurations. The green line corresponds to parallel polarization, and the red line corresponds to cross-polarization.

**Figure 8:**
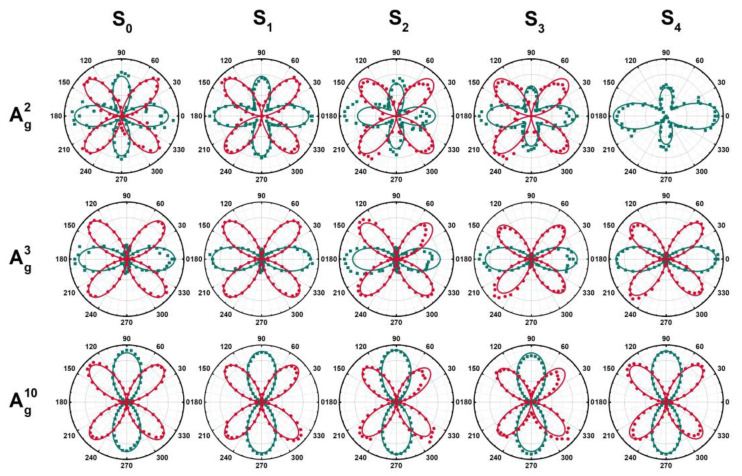
Polar diagram of ARPRS for $A_{g}^{2}$, $A_{g}^{3}$, and $A_{g}^{10}$ phonons. Solid curves correspond to the best fit of the data using Equations (9) and (10) for each configuration and mode symmetry. The green line corresponds to parallel polarization, and the red line corresponds to cross-polarization.

Based on the ARPRS results shown in Figure 7, distinct Raman phonon modes display different intensity variations with a periodic trend (similar to a sinusoidal wave). Voigt function is used to fit the $B_{g}^{2}$, $A_{g}^{2}$, and $A_{g}^{3}$ peaks and investigate their polarization dependence. The Polar diagram of Raman peak intensity as a function of the angle θ in Figure 8 exhibits clear polarization dependence. In the parallel-polarization configuration, the three modes exhibit a 2-lobed shape, where the $A_{g}^{2}$ and $A_{g}^{3}$ modes have two main lobes and two subsidiary lobes. Their major axes are oriented at 0° (180°), and the relative intensities of the subsidiary lobes decrease with increasing Al content. $A_{g}^{10}$ mode shows two dominant lobes with a main axis at 90° (270°), perpendicular to the main axes of the other two modes. In the cross-polarization configuration, all modes exhibit an almost equally sized 4-lobed shape, oriented at a 45° angle to the pattern observed under parallel polarization.

To figure out the differential polarization degree of the internal structure of the aluminum alloy sample, Equations (9) and (10) are used to fit the polar coordinates of each mode. The fitting results are shown by the green and red lines in Figure 8. The fitting curve is consistent with the experimental data. For some messy data, such as the $A_{g}^{2}$ and $A_{g}^{3}$ modes of S_2_, this may be related to the roughness of the sample surface. The (100) plane is a cleavage plane in β-Ga_2_O_3_ [24], and some cracks will occur on the surface and inside of the sample. When the test area contains cracks, the test results will be confused. Because of the lack of the $A_{g}^{2}$ mode of S_4_, the vertical polarization data cannot be effectively fitted due to the serious peak distortion. The fitted parameters are listed in Table 2. The anisotropic properties of β-(Al_x_Ga_1−x_)_2_O_3_ alloy samples on the (100) plane can be analyzed through the ratio of Raman tensor elements |a/c| and the phase difference φ. |a/c| less than 1 or greater than 1 determines whether the main axis is along the 0°/180° or 90°/270° direction, respectively. The value of |a/c| reflects the anisotropy of the Raman tensor for the A_g_ mode, and the closer it is to 1, the weaker the anisotropy. According to Table 2, it can be found that the Raman tensors of the $A_{g}^{2}$, $A_{g}^{3}$, and $A_{g}^{10}$ modes in β-(Al_x_Ga_1−x_)_2_O_3_ all exhibit significant anisotropy, but their trends with aluminum composition are different. For the pure β-Ga_2_O_3_ sample, the |a/c| values of the $A_{g}^{2}$, $A_{g}^{3}$, and $A_{g}^{10}$ modes are 0.922, 0.503, and 14.740, respectively. It corroborates that reported by Zhang et al. [41]. Relatively, $A_{g}^{10}$ exhibits very strong anisotropy, while the |a/c| value of the $A_{g}^{2}$ mode is close to 1, which suggests that the anisotropy is not significant. The anisotropy of the Raman phonon modes of β-Ga_2_O_3_ is strongly influenced by aluminum alloying. Obviously, the anisotropy of $A_{g}^{2}$ and $A_{g}^{3}$ increases with the increase of aluminum content, while the incorporation of aluminum weakens the anisotropy of $A_{g}^{10}$. This may be caused by the large difference in the bond polarizability. The β-angle increases with the increase of Al content [26], leading to the increased asymmetry of the β-(Al_x_Ga_1−x_)_2_O_3_ unit cell. Consequently, the anisotropic properties of the phonons in the low-frequency region associated with chain vibrations are strengthened. The $A_{g}^{2}$ phonon vibrates perpendicular to the ab plane [20,24], and its anisotropy is not significant when observed on the plane (100). The $A_{g}^{3}$ phonon is mainly affected by chain oscillation, and the vibration direction of the involved atoms is more diverse, resulting in a more significant anisotropy. The $A_{g}^{10}$ mode involves only the stretching of the two Ga-O bonds, resulting in a very strong anisotropy on the (100) plane. The average bond length of Al-O is shorter than that of Ga-O. When Al atoms replace Ga atoms, the polarization of the original Ga-O bonds in the (100) plane is decreased, and their anisotropy is weakened. Furthermore, Figure 7 presents an interesting phenomenon, where one can see that the relative intensity of the $A_{g}^{10}$ peak concerning the low- and medium-frequency Raman peaks undergoes significant changes with the variation of the aluminum composition, x. By taking $A_{g}^{3}$ as a reference, the strongest peak intensity of the $A_{g}^{3}$ and $A_{g}^{10}$ in all angles are selected for comparison in each data group, as shown in Table 3. It can be found that the intensity of $A_{g}^{10}$ is significantly higher than that of the $A_{g}^{3}$ after doping in both parallel and cross configurations, and the relative ratio of $A_{g}^{10}$/$A_{g}^{3}$ peak intensity becomes more and more obvious with the increase of aluminum composition. This is difficult to observe in a normal Raman spectroscopy. The ARPRS of β-(Al_x_Ga_1-x_)_2_O_3_ alloy shows significant anisotropic dependence on the polarization of the incident light. The anisotropic properties of gallium oxide cannot be ignored in different phonon modes on the plane (100) as they exhibit different behaviors in the ratio of Raman tensor elements and phase differences. The anisotropic properties make β-(Al_x_Ga_1−x_)_2_O_3_ have different properties and application potential in different directions. By studying its anisotropic properties, researchers can better design and optimize β-(Al_x_Ga_1−x_)_2_O_3_ materials and their devices to achieve specific application requirements. Studying the phonon anisotropy of β-(Al_x_Ga_1−x_)_2_O_3_ is helpful to understand the local changes in the microstructure structure of the material and is conducive to the development of potential applications of β-(Al_x_Ga_1−x_)_2_O_3_ in new optoelectronic devices.

## 4. Conclusions

In summary, with the increase of aluminum composition in β-(Al_x_Ga_1−x_)_2_O_3_, the observed Raman peaks have displayed a blue shift, and the degree of blue shift is related to the types of phonon modes. For the A_g_ phonons, the greater the contribution of O atoms to the phonon energy, the greater the influence of Al composition on the mode. Moreover, the FWHMs of β-(Al_x_Ga_1−x_)_2_O_3_ phonons broaden with the increase of aluminum composition, x, and the phonons in the low-frequency region are influenced more by the Al composition than those in the high-frequency region. After reaching the Al composition of x = 0.17, the FWHM and Al composition begin to break away from the linear relationship. Comprehensive Raman analysis by using a spatial correlation model has revealed that the CL decreases with the increase of aluminum composition, x, and the long-range order of the lattice vibration also decreases. The CL in the low-frequency region is affected more by the Al composition than in the high-frequency region. In the range of 80 K~800 K, the CL of each Raman phonon mode decreases with the increase in temperature, and the lattice disorder becomes stronger. The temperature dependence of CL decreases with the increase of Al composition and tends to become stable after Al composition increases to a certain value. The results of angle-resolved polarized Raman spectroscopy show that the different Raman peak intensities of β-(Al_x_Ga_1−x_)_2_O_3_ become highly polarization dependent. From Raman tensors, different Raman phonon modes have exhibited significant anisotropy. As the aluminum content increases, the anisotropy of phonons in the low-frequency region increases, and the anisotropy of phonons in the high-frequency region decreases. In addition, it is found that the Al doping will lead to a change in the relative ratio of the intensity of the two strongest Raman peaks in the high-frequency region and the low-frequency region of the angle-resolved polarization Raman spectrum. With the increase of aluminum content, the ratio of $A_{g}^{10}$/$A_{g}^{3}$ peak intensity increases. We strongly feel that this comprehensive study has provided meaningful results for understanding the long-range orderliness and anisotropies in technologically important β-(Al_x_Ga_1−x_)_2_O_3_ crystals.

## Figures and Tables

**Table 1:** EDS atomic percentages for S_0_, S_1_, S_2_, S_3_, and S_4_, respectively.

	S_0_ (At%)	S_1_ (At%)	S_2_ (At%)	S_3_ (At%)	S_4_ (At%)
O K	57.3	56.6	56.8	57.5	65.5
Al L	0.1	2.6	4.7	7.2	8.9
Ga L	42.4	40.8	38.5	35.3	25.5
Al/(Al + Ga)	0.00	0.06	0.11	0.17	0.26

**Table 2:** Values of Raman tensor for the $A_{g}^{2}$, $A_{g}^{3}$, and $A_{g}^{10}$ modes of β-(Al_x_Ga_1−x_)_2_O_3_ in the parallel polarization configuration.

	S_0_			S_1_			S_2_			S_3_			S_4_		
	|a/c|	φ_ac_	R^2^	|a/c|	φ_ac_	R^2^	|a/c|	φ_ac_	R^2^	|a/c|	φ_ac_	R^2^	|a/c|	φ_ac_	R^2^
$A_{g}^{2}$	0.922	0.724	0.873	0.919	0.632	0.947	0.915	0.557	0.620	0.824	0.526	0.849	0.732	0.575	0.915
$A_{g}^{3}$	0.503	0.754	0.964	0.459	0.950	0.990	0.465	0.612	0.888	0.373	0.838	0.975	0.362	1.018	0.994
$A_{g}^{10}$	14.740	0.129	0.996	14.463	0.185	0.998	13.256	0.454	0.990	12.903	0.327	0.993	11.995	0.128	0.988

**Table 3:** The peak intensity ratio of $A_{g}^{10}$/$A_{g}^{3}$ from Figure 7.

	S_0_	S_1_	S_2_	S_3_	S_4_
parallel polarization	2.129	2.322	2.462	2.815	3.147
cross-polarization	1.370	1.430	1.627	1.761	2.488

## Data Availability

The data that support the findings of this study are available from the corresponding author upon reasonable request.

## Supplementary Materials

The following supporting information can be downloaded at: https://www.mdpi.com/article/10.3390/ma16124269/s1; Figure S1: The measurement results of EDS for S_0_, S_1_, S_2_, S_3_, and S_4_, respectively; Figure S2 (a): The optical transmission spectra of S_0_, S_1_, S_2_, S_3_, and S_4_, respectively; (b) The bandgaps of the five samples calculated by using the Tauc method; Figure S3: Variable-temperature Raman spectra of S_1_, S_2_, and S_3_, respectively.
