# Supplementary material for: The Spatial Correlation and Anisotropy of β-(AlxGa1−x)2O3 Single Crystal
Source: Materials (Basel). 2023 Jun 8;16(12):4269. doi: 10.3390/ma16124269 (PMC10304312; doi:10.3390/ma16124269)
Supplement: Supplementary file 1 [file materials-16-04269-s001.zip › materials-2391182-supplementary.pdf]

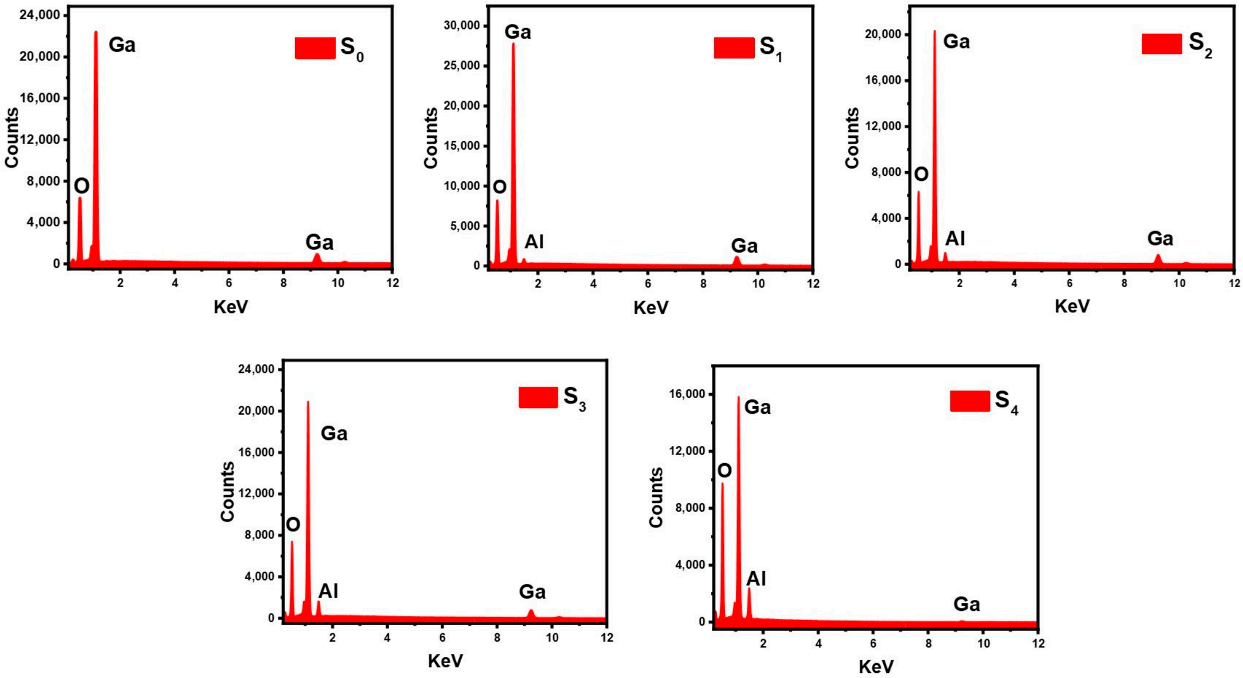

Figure S1. The measurement results of EDS for S_0, S_1, S_2, S_3, and S_4, respectively.

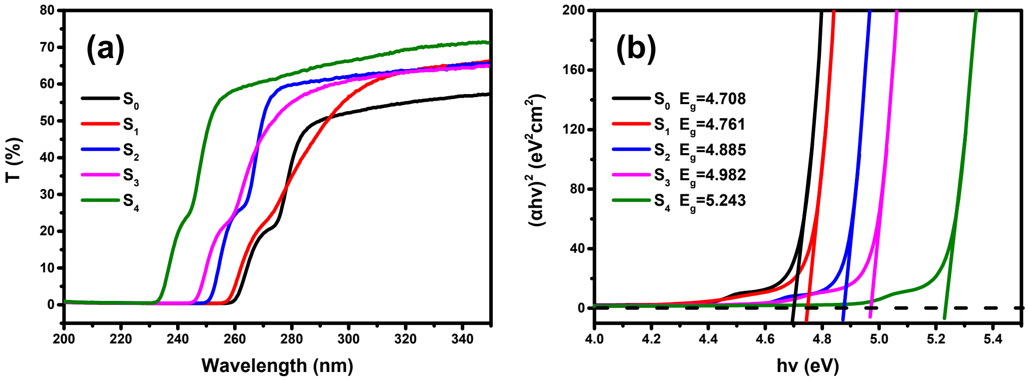

Figure S2. (a) The optical transmission spectra of S_0, S_1, S_2, S_3, and S_4, respectively; (b) The bandgaps of the five samples calculated by using Tauc method.

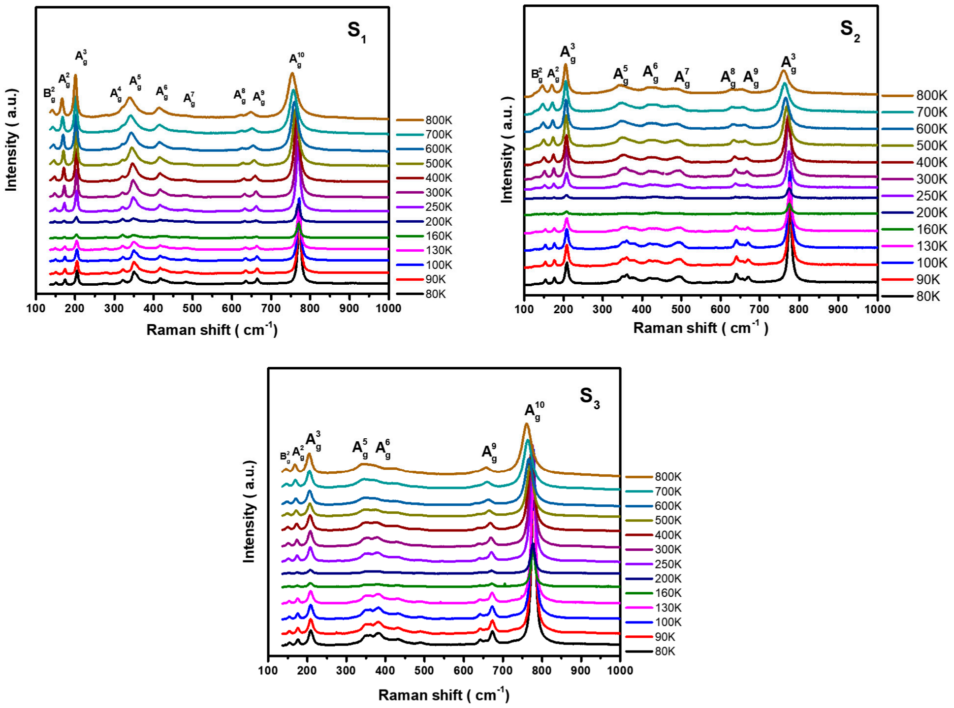

Figure S3. Variable-temperature Raman spectra of S_1, S_2, and S_3, respectively.
